# The Effect of Intravenous Thrombolysis and Mechanical Thrombectomy on Change in the Concentrations of Interleukin-18 and Degradation Products of the Endothelial Glycocalyx in Patients with Acute Ischemic Stroke

**DOI:** 10.3390/life16030387

**Published:** 2026-02-28

**Authors:** Anja Babić, Božena Ćurko-Cofek, Vlatka Sotošek, David Bonifačić, Melani Mamić, Vita Komen, Slavica Kovačić, Vladimira Vuletić, Lara Batičić

**Affiliations:** 1 Department of Neurology, Faculty of Medicine, University of Rijeka, Braće Branchetta 20, 51000 Rijeka, Croatia; anja.babic@medri.uniri.hr (A.B.); david.bonifacic@medri.uniri.hr (D.B.); melani.mamic@medri.uniri.hr (M.M.); vita.komen@medri.uniri.hr (V.K.); vladimira.vuletic@medri.uniri.hr (V.V.); 2 Department of Physiology, Immunology and Pathophysiology, Faculty of Medicine, University of Rijeka, Braće Branchetta 20, 51000 Rijeka, Croatia; 3 Department of Anesthesiology, Reanimatology, Emergency and Intensive Care Medicine, Faculty of Medicine, University of Rijeka, Braće Branchetta 20, 51000 Rijeka, Croatia; vlatkast@medri.uniri.hr; 4 Department of Clinical Medical Sciences II, Faculty of Health Studies, University of Rijeka, Rijeka, Viktora Cara Emina 2, 51000 Rijeka, Croatia; 5 Department of Radiology, Faculty of Medicine, University of Rijeka, Braće Branchetta 20, 51000 Rijeka, Croatia; slavica.kovacic@medri.uniri.hr; 6 Department of Medical Chemistry, Biochemistry and Clinical Chemistry, Faculty of Medicine, University of Rijeka, Braće Branchetta 20, 51000 Rijeka, Croatia; lara.baticic@medri.uniri.hr

**Keywords:** endothelial glycocalyx, heparan sulphate, hyaluronic acid, interleukin-18, stroke, syndecan-1, therapeutic thrombolysis, thrombectomy

## Abstract

Stroke is characterized by a sudden onset of neurological deficit attributed to a focal brain injury. The main treatments for patients with an acute ischemic stroke are intravenous thrombolysis and mechanical thrombectomy. Recanalization therapies have significantly improved patient outcomes; however, their effectiveness depends on a range of pathophysiological factors. This prospective observational study included 60 patients with acute ischemic stroke. The control group consisted of 20 healthy blood donors. Patients were divided into three groups based on whether they received intravenous thrombolysis, mechanical thrombectomy, or combination therapy. We investigated differences between recanalization therapies in patients with ischemic stroke with respect to peripheral blood concentrations of the proinflammatory cytokine interleukin (IL)-18 and endothelial glycocalyx degradation products: syndecan-1, heparan sulphate, and hyaluronic acid, measured by enzyme-linked immunosorbent assay. The blood samples were collected before, 24, and 48 h after recanalization therapy. The concentration of IL-18, syndecan-1, and heparan sulphate increased statistically significantly in patients treated with mechanical thrombectomy. The concentration of hyaluronic acid increased statistically significantly in patients treated with intravenous thrombolysis. The findings primarily reflect between-group differences. Our findings indicate that IL-18 has a significant role in the early inflammatory response. IL-18 and EG degradation products represent potential biomarkers for identifying high-risk patients. Their measurement could help improve the treatment, recovery, and outcomes in patients with acute ischemic stroke. The aforementioned observations underscore their potential value as biomarkers for future research.

## 1. Introduction

Ischemic stroke is one of the leading causes of mortality and disability worldwide [1]. It is defined as a focal brain injury resulting in a sudden-onset neurological deficit [2]. Ischemia most commonly results from thrombosis or embolism of blood vessels. Recanalization therapy for acute ischemic stroke includes intravenous thrombolysis and mechanical thrombectomy [3,4]. These therapies have significantly improved patient outcomes, but overall recovery after ischemic stroke depends on multiple factors [5,6]. Endothelial dysfunction may affect both the efficacy and safety of these treatments.

The endothelium does not function only as an anatomical barrier between blood and tissue, but also as an endocrine organ with numerous functions [7,8]. It is crucial for vascular homeostasis throughout cerebral and systemic circulation, and its disruption is linked to hypertension, atherosclerosis, and other cardiovascular disorders [9]. Endothelial cells are covered on their luminal surface by the endothelial glycocalyx (EG), a gel-like layer of glycosylated proteins that prevents direct contact between blood components and the vessel wall.

The EG is a dynamic structure produced by endothelial cells that undergoes continuous degradation and restoration in response to mechanical and biochemical stimuli. Extending into the vessel lumen, the EG transduces shear stress into mechanical signals that activate endothelial nitric oxide synthase (eNOS), thereby promoting nitric oxide (NO)-mediated vasodilation [10]. The EG is composed mainly of proteoglycans and glycoproteins, with attached glycosaminoglycan (GAG) chains, which provide structural support. Key proteoglycans include syndecans, particularly syndecan-1, and glypicans. Syndecan-1 is widely studied for its roles in cell behavior, inflammation, and mechanotransduction [11]. Major GAGs are heparan sulphate, which constitutes 50–90% of total EG GAG content, and hyaluronic acid, a non-sulfated polysaccharide that contributes to barrier function via CD44 [12,13,14]. Sulphated GAGs and sialic acid-containing glycoproteins confer the negative charge of the EG, thereby regulating vascular permeability and limiting cellular adhesion.

Endothelial function varies across the arterial system, with the cerebral endothelium being highly specialized to form and maintain the blood–brain barrier (BBB), a key component of the neurovascular unit that preserves the brain’s microenvironment. Cerebral endothelial cells are characterized by tight junctions, absence of fenestrations, low pinocytotic activity, and specialized enzymatic functions [5]. Endothelial dysfunction is marked by reduced NO bioavailability, impaired endothelium-dependent vasodilation, and a shift toward a proinflammatory and procoagulant state [15]. This promotes inflammatory mediator release, including IL-18, a proinflammatory cytokine involved in innate and adaptive immunity that has been increasingly implicated in ischemic stroke and atherosclerotic plaque instability [16]. IL-18 is encoded by the human IL-18 gene and is mainly produced by macrophages and mononuclear cells, but also by several other cell types, including activated microglia, astrocytes, and ependymal cells [17]. IL-18 is synthesized as an inactive precursor, pro-IL-18, which is subsequently cleaved into its biologically active form through proteolytic processing, predominantly by caspase-1 [18].

Restoration of cerebral blood flow to previously ischemic tissue may initiate reperfusion-related injury, a process driven by oxidative stress and inflammatory activation. Endothelial dysfunction can amplify this response by promoting the BBB disruption, increasing reactive oxygen species production, and enhancing inflammatory signaling, thereby extending tissue damage beyond the point of successful vessel reopening. Hemorrhagic transformation occurs more frequently after recanalization therapies and is associated with poorer stroke outcomes. Intravenous thrombolysis restores blood flow through enzymatic clot dissolution. Alteplase converts plasminogen to plasmin, which not only degrades fibrin but also activates matrix metalloproteinases (MMPs) [19]. This process can lead to degradation of extracellular matrix components and shedding of the EG, compromising endothelial barrier integrity and promoting inflammation. In addition, thrombolysis-associated reperfusion enhances microglial and macrophage activation within the neurovascular unit, which are key sources of IL-18 [18]. Mechanical thrombectomy has been linked to BBB injury, suggesting that direct mechanical interaction with the endothelium contributes to procedure-related vascular damage. This localized vascular trauma, combined with abrupt changes in reperfusion dynamics, may stimulate IL-18 release from activated endothelial cells and infiltrating immune cells at the site of intervention. Impaired NO bioavailability compromises vasodilatory capacity, potentially reducing the hemodynamic benefit of recanalization therapies, as adequate NO signaling is essential for effective reperfusion of ischemic regions. Moreover, endothelial dysfunction is associated with a prothrombotic environment that predisposes to reocclusion following recanalization. The dynamics of reperfusion also differ between modalities, influencing the magnitude of reperfusion injury, endothelial activation, and inflammatory response. Rapid reperfusion may exacerbate inflammatory cascades, whereas delayed or incomplete recanalization prolongs ischemic endothelial damage [20].

Considering the above, it is necessary to investigate how endothelial dysfunction and the inflammatory response contribute to stroke progression, to identify targets for novel therapies aimed at modulating the immune response and thereby reducing ischemia-induced brain injury. The aim of this study was to analyze the differences between recanalization therapies in acute ischemic stroke patients by comparing peripheral blood levels of proinflammatory IL-18 and EG degradation products, as measured by enzyme-linked immunosorbent assay (ELISA) in the peripheral circulation.

## 2. Materials and Methods

### 2.1. Patients

This prospective observational study included 60 patients with acute ischemic stroke admitted to the Department of Neurology at the University Hospital Rijeka, Rijeka, Croatia, between October 2023 and September 2025. The protocol was approved by the Ethics Committee of the Clinical Hospital Centre Rijeka and the Committee of the Faculty of Medicine Rijeka, University of Rijeka, Rijeka, Croatia, and was carried out according to the World Medical Association criteria in the Declaration of Helsinki. All patients or their family members were provided with an information form and additional explanations from the researchers involved in the study. Patients were enrolled in the study after providing written informed consent.

The patients with acute ischemic stroke were divided into three groups based on the treatment modality. Each group included 20 patients. Group 1 patients received intravenous thrombolysis alone, Group 2 patients were treated with intravenous thrombolysis and mechanical thrombectomy, and Group 3 patients underwent mechanical thrombectomy alone. Patients treated with intravenous thrombolysis were administered alteplase. The choice of therapy was in accordance with the guidelines of the European Stroke Organization [3,4]. Patients younger than 18 and those with confirmed autoimmune disease, malignant disease, and infection, as well as those who were on anticoagulation therapy, were excluded from the study. The control group consists of 20 healthy blood donors, 12 male and 8 female, aged 58–65 years, selected based on the absence of cardiovascular risk factors.

Demographic and clinical parameters were recorded, including age, sex, length of hospital stay, and outcome. Stroke severity was evaluated using the National Institutes of Health Stroke Scale (NIHSS), and functional outcomes were assessed with the modified Rankin Scale (mRS). NIHSS scores were obtained at admission, at discharge, and at the 90-day follow-up after stroke onset. The mRS was documented at discharge and reassessed 90 days after stroke onset.

### 2.2. Blood Collection

Ten milliliters of peripheral venous blood were collected in anticoagulant-free blood collection tubes (Vacuette Tube, Greiner Bio-One, Kremsmünster, Austria) from each study participant. Peripheral venous blood samples were obtained immediately prior to recanalization therapy (T1) and at 24 h (T2) and 48 h (T3) following the therapy, and left to clot at room temperature for 1 h. Serum was collected by centrifugation at 3500× *g* for 10 min and stored at −80 °C until further analysis.

### 2.3. Determination of the Serum Concentration of IL-18, Syndecan-1, Heparan Sulphate and Hyaluronic Acid

Serum levels of IL-18, syndecan-1, heparan sulphate, and hyaluronic acid were measured using high-sensitivity ELISAs obtained from ELK Biotechnology (Denver, CO, USA), according to the manufacturer’s protocols. Serum samples and standards were added to antibody-precoated microplate wells. Following incubation and washing to remove unbound components, a biotin-labeled detection antibody was added, followed by streptavidin–horseradish peroxidase. The colorimetric reaction was developed using TMB substrate and terminated with the supplied acidic solution. Optical density was measured at 450 nm with a reference wavelength of 630 nm using a microplate reader (EL808, BioTek Instruments, Winooski, VT, USA). Analyte concentrations were determined from standard curves and expressed in pg/mL for IL-18 and heparan sulphate, and in ng/mL for syndecan-1 and hyaluronic acid. Data analysis was performed using the online software available at https://www.myassays.com/ (accessed on 20 November 2025) (Brighton, East Sussex, UK).

### 2.4. Statistical Analysis

Statistical analyses were performed using Statistica software version 14.0.0 (TIBCO Software Inc., Palo Alto, CA, USA). Data distribution was assessed with the Kolmogorov–Smirnov test. Non-normally distributed data were analyzed using non-parametric methods. Within-group comparisons over time were performed using Friedman’s test, followed by the Wilcoxon signed-rank test for post hoc analysis. Between-group differences were evaluated with the Kruskal–Wallis test, followed by the Mann–Whitney U-test for post hoc comparisons, with Bonferroni correction applied for multiple testing, and the adjusted significance threshold was *p* < 0.025. Sample size calculation was performed using G*Power software (version 3.1.9.7; Heinrich Heine University Düsseldorf, Düsseldorf, Germany) based on serum IL-18 values reported in previous studies [18,21]. The calculation assumed a type I error rate of 0.05 and a type II error rate of 0.20 (80% statistical power). Based on these parameters, the required number of participants was estimated to be 20 per treatment group. Correlations were assessed using Spearman’s rank correlation coefficient. Categorical variables were analyzed with the chi-square test or Fisher’s exact test, as appropriate, and a *p*-value < 0.05 was considered statistically significant. Data are presented as 25th–75th percentile values.

## 3. Results

### 3.1. Patient Demographics and Clinical Data

The demographic and clinical data of patients with stroke who were treated with recanalization therapies are presented in Table 1. Patients did not differ in age, length of hospitalization, or outcome. Although the difference is not statistically significant, patients in Group 3 exhibited a broader dispersion in length of hospital stay, which could be influenced by variability in clinical presentation or treatment-related factors.

The patients differed in terms of sex: Groups 1 and 3 were predominantly male, whereas Group 2 had an equal representation of both sexes.

The NIHSS score at admission, the mRS score at discharge, and at 90-day follow-up did not differ among groups (Table 1).

Two patients from each Group 2 and Group 3 were lost at 90-day follow-up because of the hemorrhagic transformation that resulted in death.

Group 2 and Group 3 patients who underwent mechanical thrombectomy achieved complete reperfusion, with a Thrombolysis in Cerebral Infarction (TICI) score of 3.

Only one patient from Group 3 had a large vessel occlusion in the posterior circulation, whereas all remaining patients from Groups 2 and 3 had large vessel occlusions in the anterior circulation.

### 3.2. The Influence of Recanalization Therapies on the Concentration of IL-18

**Figure 1:**
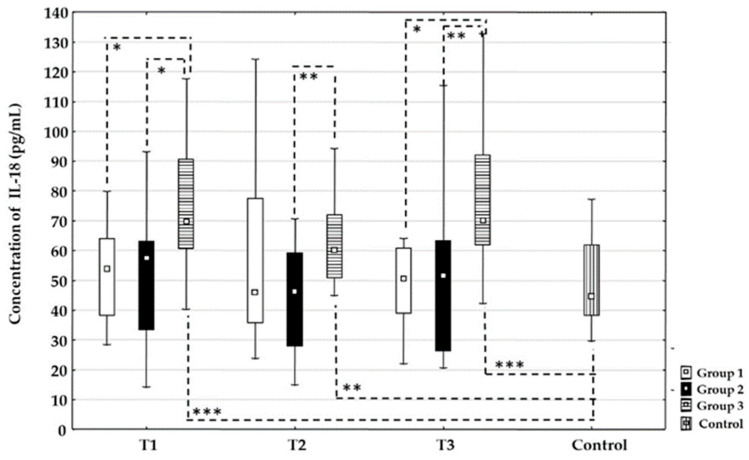
Time-related changes in interleukin-18 (IL-18) concentration in different recanalization therapies after acute ischemic stroke. Dynamic changes and comparison of the IL-18 concentration among Group 1 treated with intravenous thrombolysis (□), Group 2 treated with intravenous thrombolysis and mechanical thrombectomy (■), Group 3 treated with mechanical thrombectomy (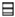) and the control group (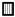) before recanalization therapy (T1), at 24 h (T2), and at 48 h (T3) after recanalization therapy. Level of statistical significance: *, *p* ≤ 0.025; **, *p* ≤ 0.01; and ***, *p* ≤ 0.001. Data are expressed as median and 25th–75th percentile.

The influence of recanalization therapies on the concentration of IL-18 is shown in Figure 1. There were no statistically significant changes in the concentration of IL-18 between time points T1, T2, or T3 for each treatment group. At time point T1, the concentration values of IL-18 were statistically higher in Group 3 compared to Group 1 (*p* = 0.023) and Group 2 (*p* = 0.021), while there was no difference between Groups 1 and 2. At time point T2, the concentration values of IL-18 were significantly higher in Group 3 compared to Group 2 (*p* = 0.003). There were no differences between Groups 1 and 2 and between Groups 1 and 3. At time point T3, the concentration values of IL-18 were statistically higher in Group 3 compared to Group 1 (*p* = 0.015) and Group 2 (*p* = 0.008). There were no differences between Groups 1 and 2. Additionally, there was no difference in IL-18 concentration between Groups 1 and 2 and the control group at all time points. In contrast, Group 3 had significantly higher concentrations of IL-18 at all time points compared to the control group: T1 (*p* < 0.001), T2 (*p* = 0.006), T3 (*p* < 0.001).

### 3.3. The Influence of Recanalization Therapies on EG Degradation Products

#### 3.3.1. Syndecan-1

**Figure 2:**
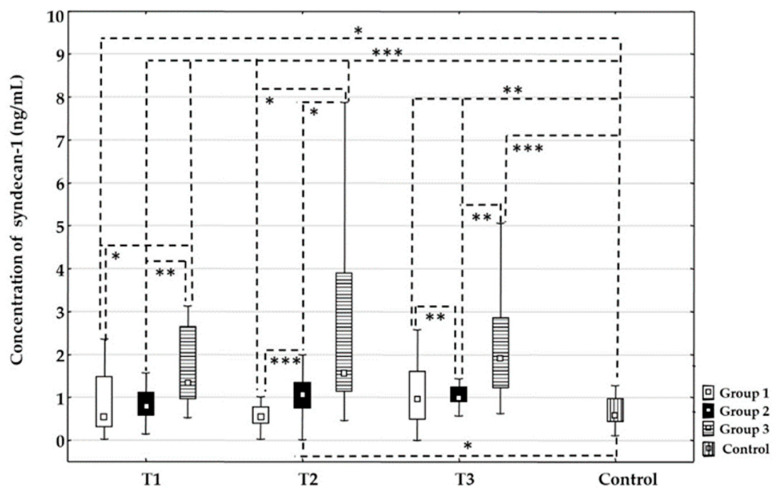
Time-related changes in syndecan-1 concentration in different recanalization therapies after acute ischemic stroke. Dynamic changes and comparison of the concentration of syndecan-1 between the Group 1 treated with intravenous thrombolysis (□), Group 2 treated with intravenous thrombolysis and mechanical thrombectomy (■), Group 3 treated with mechanical thrombectomy (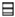), and the control group (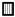) before recanalization therapy (T1), at 24 h (T2), and at 48 h (T3) after recanalization therapy. Level of statistical significance: *, *p* ≤0.025; **, *p* ≤ 0.01; and ***, *p* ≤ 0.001. Data are expressed as median and 25th–75th percentile.

There were no statistically significant changes in the concentration of syndecan-1 between time points T1, T2, or T3 for each treatment group. At time point T1, the concentration values of syndecan-1 were statistically higher in Group 3 compared to Group 1 (*p* = 0.020) and Group 2 (*p* = 0.01). In addition, there were no differences between Groups 1 and 2 at time point T1. At time point T2, the concentration values of syndecan-1 were significantly higher in Group 3 compared to Groups 1 (*p* < 0.001) and 2 (*p* = 0.023), and significantly higher in Group 2 compared to Group 1 (*p* < 0.001). At time point T3, the concentration values of syndecan-1 were significantly higher in Group 2 compared to Group 1 (*p* = 0.005) and significantly higher in Group 3 compared to Group 2 (*p* = 0.004). In addition, there were no differences between Groups 1 and 3 at time point T3. The concentration values of syndecan-1 were significantly higher in all time points for all groups compared to the control group: Group 1 T1 (*p* = 0.020), T2 (*p* = 0.001), T3 (*p* < 0.01); Group 2 T1 (*p* = 0.001), T2 (*p* = 0.013), T3 (*p* = 0.01); and Group 3 T1 (*p* < 0.001), T2 (*p* = 0.001), T3 (*p* < 0.001), respectively (Figure 2).

#### 3.3.2. Heparan Sulphate

**Figure 3:**
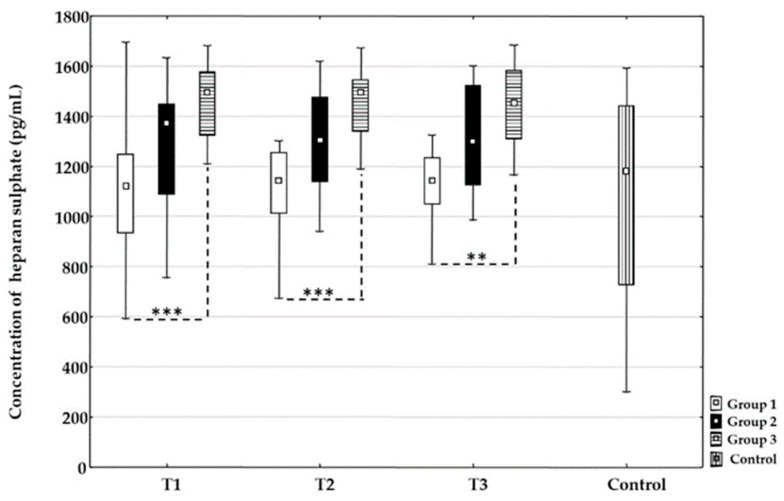
Time-related changes in heparan sulphate concentration in different recanalization therapies after acute ischemic stroke. Dynamic changes and comparison of the concentration of heparan sulphate between the Group 1 treated with intravenous thrombolysis (□), Group 2 treated with intravenous thrombolysis and mechanical thrombectomy (■), Group 3 treated with mechanical thrombectomy (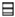) and the control group (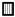) before recanalization therapy (T1), at 24 h (T2), and at 48 h (T3) after recanalization therapy. Level of statistical significance: **, *p* ≤ 0.01; ***, *p* ≤ 0.001. Data are expressed as median and 25th–75th percentile.

There were no statistically significant changes in the concentration of heparan sulphate between time points T1, T2, or T3 for each treatment group. At all time points, the concentration values of heparan sulphate were statistically higher in Group 3 compared to Group 1: T1 (*p* = 0.001), T2 (*p* = 0.001), T3 (*p* = 0.002). There were no differences between Groups 1 and 2 and between Groups 2 and 3 at all time points. In addition, there were no differences between all groups compared to the control group at all time points (Figure 3).

#### 3.3.3. Hyaluronic Acid

**Figure 4:**
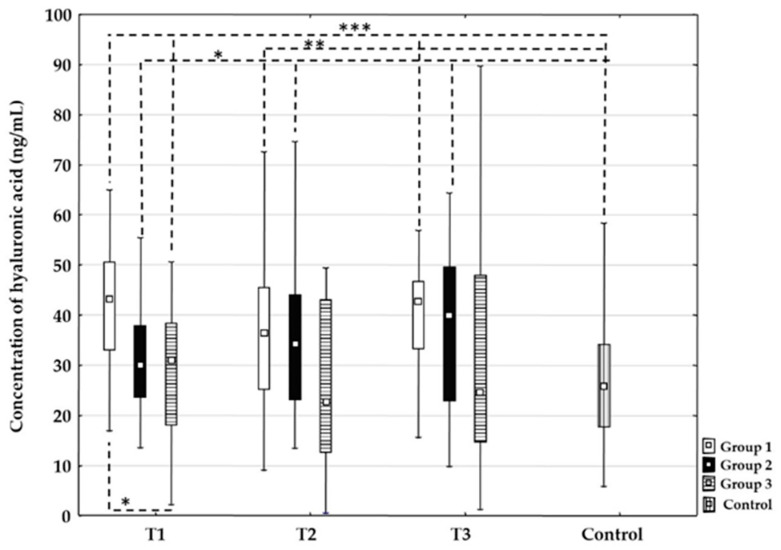
Time-related changes in hyaluronic acid concentration in different recanalization therapies after acute ischemic stroke. Dynamic changes and comparison of the concentration of hyaluronic acid between the Group 1 treated with intravenous thrombolysis (□), Group 2 treated with intravenous thrombolysis and mechanical thrombectomy (■), Group 3 treated with mechanical thrombectomy (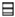) and the control group (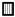) before recanalization therapy (T1), and 24 h (T2) and 48 h (T3) after recanalization therapy. Level of statistical significance: * *p* ≤0.025, ** *p* ≤ 0.01, *** *p* ≤ 0.001. Data are expressed as median and 25th–75th percentile.

There were no statistically significant changes in the concentration of hyaluronic acid between time points T1, T2, or T3 for each treatment group. At time point T1, the concentration values of hyaluronic acid were statistically higher in Group 1 compared to Group 3 (*p* = 0.016). In addition, there were no differences between Groups 1 and 2 and Groups 2 and 3 at time point T1. At time points T2 and T3, there were no significant differences between all groups. The concentration values of hyaluronic acid were significantly higher in time point T1 for all groups compared to the control group: Group 1 (*p* < 0.001); Group 2 (*p* = 0.023); and Group 3 (*p* = 0.001), respectively. In addition, at time point T2, the values were higher in Group 1 (*p* = 0.003) and Group 2 (*p* = 0.013) compared to the control group, and at time point T3, the values were higher in Group 1 (*p* = < 0.001) and Group 2 (*p* = 0.020) (Figure 4).

### 3.4. Correlations Between the Serum Concentration of IL-18 and EG Degradation Products

The correlations between the concentration of IL-18 and the concentration of syndecan-1, heparan sulphate, and hyaluronic acid are shown in Table 2. In Group 1, a negative correlation was found between the concentration of IL-18 and the concentration of hyaluronic acid at time point T3 (r = −0.606, *p* = 0.005). A positive correlation was observed between the concentration of IL-18 and the concentration of hyaluronic acid at time points T1 (r = 0.605, *p* = 0.005), T2 (r = 0.602, *p* = 0.005) and T3 (r = 0.742, *p* = 0.000) in the Group 2 and time point T3 in the Group 3 (r = 0.451, *p* = 0.046). A positive correlation was observed between the concentration of IL-18 and the concentration of heparan sulphate at time points T1 (r = 0.615, *p* = 0.004), T2 (r = 0.617, *p* = 0.004) and T3 (r = 0.554, *p* = 0.011) in the Group 1 and between the concentration of IL-18 and the concentration of syndecan-1 at time point T1 in the Group 1 (r = 0.507, *p* = 0.022).

### 3.5. Correlations Between the Serum Concentration of IL-18 and EG Degradation Products, NIHSS, and mRS

The correlations between the concentration of IL-18, syndecan-1, heparan sulphate, hyaluronic acid, NIHSS score, and mRS are shown in Table 3, Table 4, Table 5 and Table 6. A positive correlation was observed between the concentration of IL-18 and NIHSS score at admission in Group 2 in all time points (T1 r = 0.520, *p* = 0.025; T2 r = 0.544, *p* = 0.020; T3 r = 0.565, *p* = 0.018) (Table 3), as well as between the concentration of hyaluronic acid and NIHSS at admission (r = 0.600, *p* = 0.005) and at 90-day follow-up (r = 0.493; *p* = 0.027) in Group 1 at time point T3 (Table 6). The concentrations of syndecan-1 did not show any correlation, positive or negative, with NIHSS score or mRS (Table 4). Negative correlation was observed between the concentration of heparan sulphate and NIHSS at admission in Group 1 at time points T1 (r = −0.599, *p* = 0.005) and T2 (r = −0.455, *p* = 0.044), between the concentration of heparan sulphate and NIHSS at discharge in Group 1 at all time points (T1 r = −0.564, *p* = 0.01; T2 r = −0.598, *p* = 0.005; T3 r = −0.629, *p* = 0.003) and between the concentration of heparan sulphate and NIHSS at 90-day follow-up in Group 1 at all time points (T1 r = −0.600, *p* = 0.005; T2 r = −0.635, *p* = 0.003; T3 r = −0.655, *p* = 0.002). Negative correlation was also observed between the concentration of heparan sulphate and mRS at discharge in Group 1 at time points T2 (r = −0.450, *p* = 0.047) and T3 (r = −0.456, *p* = 0.043), between the concentration of heparan sulphate and mRS at 90-day follow-up in Group 1 at all time points (T1 r = −0.535, *p* = 0.015; T2 r = −0.532, *p* = 0.016; T3 r = −0.514, *p* = 0.020) (Table 5).

## 4. Discussion

### 4.1. Stroke and IL-18

Stroke is characterized by a cascade of neuropathological events in which a strong and sustained inflammatory response plays a central role in the progression of brain injury. Post-ischemic inflammation is closely linked to the acute disruption of the BBB, contributing to worse neurological outcomes [22]. Following cerebral ischemic injury, various inflammatory cells become activated. As a part of the inflammatory reaction, necrotic tissue is removed by coordinated cellular, humoral, and metabolic mechanisms [23]. In the acute stage of cerebral infarction, ischemic and hypoxic conditions activate leukocytes, which adhere to the vessel wall and release reactive oxygen species, thereby worsening tissue damage. Brain tissue necrosis in acute ischemic stroke provokes a systemic inflammatory response, reflected by increased peripheral inflammatory indicators. A wide array of cytokines is involved in ischemic stroke, functioning with both pro-inflammatory and anti-inflammatory effects. Pro-inflammatory mediators, including IL-18, promote the progression of stroke and intensify neuroinflammation [24].

Our results showed that the serum concentration of IL-18 was statistically significantly higher at all time points in the group treated with mechanical thrombectomy compared to the group treated with a combination of recanalization therapies and the control group. Also, the serum concentration of IL-18 was statistically significantly higher at time points T1 and T3 in the group treated with mechanical thrombectomy compared to the group treated with intravenous thrombolysis alone. These results suggest an association between intravenous thrombolysis and differences in the inflammatory response, consistent with earlier findings [25,26,27]. Earlier studies have shown that alteplase can suppress inflammation, thereby decreasing the likelihood of hemorrhagic transformation and lowering post-thrombolysis bleeding risk [28]. One possible explanation is that alteplase may modulate the release of inflammatory mediators, which could potentially limit inflammation-related tissue damage and support neurological recovery.

Mechanical thrombectomy alone leads to higher IL-18 levels due to potential direct mechanical injury to the endothelium. It can cause localized vascular injury that promotes the release of IL-18 from endothelial cells and infiltrating immune cells. The abrupt reperfusion dynamics following mechanical thrombectomy may intensify ischemia–reperfusion injury. Sudden restoration of flow can enhance oxidative stress and inflammasome activation, key drivers of IL-18 maturation and release. Mechanical thrombectomy is often performed in patients with more severe strokes due to large vessel occlusion. They are associated with potential extensive BBB disruption and heightened neuroinflammatory responses.

Although restoring blood flow is necessary to save tissue, potential reperfusion injury remains a major challenge in acute stroke treatment. Current research focuses on targeting these inflammation-driven mechanisms to achieve better clinical outcomes after reperfusion interventions.

### 4.2. Stroke and EG

The breakdown of the BBB is a key pathological driver of cerebral edema and hemorrhagic transformation and has therefore attracted considerable research interest [29]. Damage to the EG appears to be an early, initiating event in the disruption of the BBB [30,31]. Earlier studies have demonstrated that elevated concentrations of EG constituents can be found in the plasma of patients with acute stroke [19,32]. Our results showed statistically higher values of syndecan-1 in all groups and at all time points compared to the control group. This suggests that stroke itself causes endothelial degradation regardless of the recanalization therapy. In addition, our results showed that the concentration of syndecan-1 was statistically higher in the group treated with mechanical thrombectomy compared to the group treated with intravenous thrombolysis and the group treated with a combination of these therapies. These results suggest that administration of thrombolysis alone or prior to mechanical thrombectomy results in less EG damage compared with mechanical thrombectomy alone. These findings are consistent with other research and suggest that a thrombolytic agent may partially inhibit syndecan-1 shedding, thereby helping to preserve EG integrity [33]. Elevated circulating syndecan-1 may also serve as an indicator of glycocalyx breakdown [34]. The influence of alteplase on syndecan-1 shedding is probably indirect. Thrombolytic agents exert their fibrinolytic action by promoting the conversion of plasminogen into plasmin, which subsequently breaks down fibrin and inactivates several coagulation components, including factor V. Factor V supports thrombin formation through the prothrombinase complex with factor X. Thrombin further promotes the release of heparanase, which degrades endothelial heparan sulphate and increases syndecan-1 shedding [21]. Heparanase is an endoglycosidase that selectively degrades heparan sulphate chains, thereby promoting increased syndecan-1 shedding. Based on a broad review of the literature, we propose that thrombolytic agents may reduce syndecan-1 shedding by modulating the thrombin–heparanase pathway [35,36].

Our results showed that the concentration of heparan sulphate was statistically higher in the group treated with mechanical thrombectomy compared to the group treated with intravenous thrombolysis at all time points. Considering the data related to syndecan-1, we can hypothesize a similar underlying mechanism. For hyaluronic acid, values were significantly higher in all groups at T1 and remained higher in the thrombolysis and combined groups through T2 and T3 compared to the control group, suggesting that the increase was likely driven by the applied recanalization therapy rather than stroke etiology. Additionally, there were no dynamic changes in any of the three groups, indicating that values remained stable throughout the examined period. When comparing the groups at all time points, the only observed difference was at T1 between the thrombolysis group and the thrombectomy group, which might suggest that microcirculation occlusion could induce higher hyaluronic acid degradation compared to occlusion of a larger vessel. From time point T2 to T3, there were no differences between any of the groups. Since no dynamic changes were observed in any groups, a larger sample size or different time points might provide a better understanding of changes in hyaluronic acid concentration in patients undergoing recanalization therapy.

Heparan sulphate values showed no difference in different time points between all recanalization procedure samples and the control group, indicating it was relatively stable in patients affected by stroke. In addition, our results showed a significant difference in values between the thrombectomy group and the thrombolysis group, suggesting that either thrombolytic therapy had a protective effect on heparan sulphate or that the shear stress and mechanical effects of reperfusion with the thrombectomy procedure had a more disruptive impact. There were no significant differences between the combined group and the other two groups, but the values were intermediate, which may also support the aforementioned interpretations. These findings suggest distinct patterns of EG injury, likely reflecting differences in mechanical versus enzymatic reperfusion mechanisms. Elevated circulating heparan sulphate in thrombectomy-treated patients may be associated with acute mechanical disruption of the endothelial surface layer, caused by direct instrument–endothelium interaction, high shear stress during clot retrieval, and abrupt reperfusion-induced oxidative stress. Mechanical thrombectomy may therefore preferentially induce shedding of structurally bound glycocalyx components, particularly heparan sulphate. In contrast, higher hyaluronic acid concentrations found in patients treated with intravenous thrombolysis may be associated with a biochemically mediated endothelial response, which may promote enzymatic degradation of hyaluronic acid.

### 4.3. Diagnostic, Prognostic, and Potential Therapeutic Implications of Studied Biomarkers

EG degradation reflects loss of barrier integrity and altered microcirculatory regulation, processes that may contribute to secondary brain injury even after successful reperfusion [37]. Our results show a statistically significant negative correlation at time point T3 between IL-18 and hyaluronic acid in the thrombolytic group. Alteplase can suppress inflammation and might lower IL-18; on the other hand, it dissolves the blood clots that caused the stroke. After clot lysis, there is a rise in circulating hyaluronic acid, which explains our observed negative correlation. In the combined group, we observed a statistically significant positive correlation at all time points between IL-18 and hyaluronic acid, probably because of the combined effect of mechanical stress and thrombolytic therapy on the blood clot. In the thrombectomy group, we observed a positive correlation only at the T3 time point, which might indicate that the effects of mechanical stress and reperfusion injury took longer to become apparent. When examining the relationship between IL-18 and heparan sulphate, a positive correlation was observed at all measured time points in the thrombolysis-treated group. Prior to treatment, this association may reflect concurrent increases in inflammatory activity, as indicated by IL-18, and heparan sulphate release as part of the endothelial antithrombotic response. After initiation of thrombolytic therapy, the strength of this positive correlation decreased, which may suggest progressive utilization or depletion of heparan sulphate during clot dissolution and ongoing endothelial activation. Regarding syndecan-1, we observed a positive correlation in the thrombolytic group at time point T1, which means the inflammatory response prior to therapy might have induced further EG shedding and produced a rise in syndecan-1 in circulation. These biomarkers may hold value for future risk stratification and outcome prediction. The combined assessment of these biomarkers may therefore provide complementary information on inflammatory and endothelial responses that are not captured by standard clinical parameters alone.

Our results showed a positive correlation between IL-18 and NIHSS score at admission in the group treated with intravenous thrombolysis and mechanical thrombectomy, indicating that higher systemic inflammatory activity is associated with greater neurological deficit.

A negative correlation was found in the group treated with thrombolysis between the concentration of heparan sulphate, NIHSS score (at admission, discharge, and 90-day follow-up), and mRS (at discharge and 90-day follow-up). Although higher serum heparan sulphate reflects more extensive EG damage, we observed a negative correlation with NIHSS and mRS in thrombolysis patients. This observation should be interpreted with caution. This paradox may be explained by dynamic changes in heparan sulphate levels after ischemia and reperfusion, including rapid clearance or consumption in patients with the most severe strokes. The timing of measurement relative to stroke onset may also influence heparan sulphate concentrations. Thus, while elevated heparan sulphate indicates glycocalyx disruption, its association with neurological outcomes may be modulated by stroke severity, endothelial exhaustion, and systemic regulatory mechanisms in these patients. Furthermore, a positive correlation was identified between serum hyaluronic acid concentrations and NIHSS scores both at admission and at the 90-day follow-up among patients treated with thrombolysis. Even with successful thrombolysis, the initial ischemic injury and EG disruption have already occurred. Therefore, hyaluronic acid levels at admission may indicate the extent of vascular and tissue damage prior to reperfusion therapy, explaining the correlation with NIHSS score at admission. While thrombolysis may improve recanalization and limit additional injury, persistent inflammation can impair neural repair and functional recovery, contributing to higher NIHSS scores at 90-day follow-up despite thrombolysis. From a therapeutic perspective, the observed associations highlight inflammatory and endothelial pathways that may be amenable to targeted intervention. IL-18-mediated signaling and glycocalyx degradation represent potentially modifiable mechanisms involved in ischemia–reperfusion injury. Further experimental and clinical studies are required to determine whether modulation of these pathways translates into improved microvascular function and neurological outcomes.

### 4.4. Future Perspectives of IL-18 and EG in Acute Stroke

Growing evidence supports the important role of IL-18 as a pro-inflammatory cytokine and the integrity of the EG in acute ischemic stroke. IL-18 promotes neuroinflammation, tissue damage, and secondary vascular injury, while disruption of the EG worsens endothelial dysfunction, compromises the BBB, and amplifies inflammatory responses [38,39]. Circulating IL-18 and EG-derived components, such as heparan sulphate and hyaluronic acid, may serve as early prognostic biomarkers for identifying patients at risk of severe deficits or poor recovery. Repeated measurement of these markers could provide insight into the efficacy of recanalization therapies. Therapeutic strategies aimed at modulating IL-18 signaling or protecting the EG may offer novel neuroprotective approaches, potentially reducing infarct size, limiting inflammation, and improving functional outcomes.

### 4.5. Study Limitations

This study has several limitations that should be considered when interpreting the results. The relatively small sample size limits the generalizability of the findings; therefore, confirmation in larger, multicenter cohorts is warranted. In addition, the limited number of sampling time points may have overlooked relevant fluctuations in inflammatory and endothelial responses during the acute phase of stroke. The exact time of symptom onset could not be reliably determined for all patients, particularly in unwitnessed or wake-up strokes, which represents a limitation of this study. Limiting the analysis to patients with complete reperfusion (TICI 3) may restrict the generalizability of the findings. More frequent biomarker measurements would allow a more detailed assessment of temporal dynamics. The low proportion of female participants may limit the applicability of the findings across sexes. Incorporating additional inflammatory mediators alongside comprehensive markers of endothelial dysfunction would provide a more complete understanding of the pathophysiological mechanisms involved in acute ischemic stroke.

## 5. Conclusions

IL-18 plays a central role in the inflammatory response following acute ischemic stroke, contributing to endothelial dysfunction, BBB disruption, and secondary vascular injury. EG degradation, reflected by circulating components such as heparan sulphate and hyaluronic acid, further exacerbates vascular and tissue damage. Monitoring these biomarkers in patients undergoing recanalization therapies may provide valuable prognostic information and help identify patients at higher risk of secondary vascular complications. Furthermore, targeting IL-18–mediated inflammatory pathways or protecting the EG represents a promising strategy to reduce reperfusion-associated injury and improve neurological recovery. Overall, integrating IL-18 and glycocalyx assessment into clinical practice could support personalized treatment approaches and guide the development of novel neuroprotective interventions in acute stroke care.

## Figures and Tables

**Table 1:** Basic demographic and clinical data of the patients.

	Group 1 (*n* = 20)	Group 2 (*n* = 20)	Group 3 (*n* = 20)	*p* Value
**Age (years)**	71.5 (64.5–75)	69.5 (66–77.5)	68 (64.5–74)	0.674
**Sex (male/female)**	17/3	10/10	15/5	0.045
**Length of hospital stay (days)**	6 (5–8.5)	6 (5–8)	8 (5.5–20)	0.094
**Outcome** **(discharged/death)**	20/0	18/2	18/2	0.803
**NIHSS at admission**	5 (4–7)	6 (4–11)	7 (5–13)	0.150
**mRS at discharge**	3 (2–4)	3 (2–5)	8 (2–10)	0.065
**mRS 90 days**	2 (2–4.5)	2 (1–4)	4 (2–9)	0.220

Continuous variables are presented as median and 25th–75th percentiles, and categorical data are presented as the number of cases (*n*). Group 1—patients who received intravenous thrombolysis alone; Group 2—patients who were treated with intravenous thrombolysis and mechanical thrombectomy; Group 3—patients who underwent mechanical thrombectomy alone.

**Table 2:** Correlations between the concentration of IL-18 and concentrations of syndecan-1, heparan sulphate, and hyaluronic acid.

Correlation	Concentration of IL-18 (pg/mL)																	
	Group 1						Group 2						Group 3					
	T1		T2		T3		T1		T2		T3		T1		T2		T3	
	r	*p*	r	*p*	r	*p*	r	*p*	r	*p*	r	*p*	r	*p*	r	*p*	r	*p*
Concentration of syndecan-1 (ng/mL)	**0.507**	**0.022**	0.043	0.856	−0.086	0.719	−0.195	0.409	−0.400	0.081	−0.390	0.099	0.040	0.867	0.171	0.471	−0.293	0.210
Concentration of heparan sulphate (pg/mL)	**0.615**	**0.004**	**0.617**	**0.004**	**0.554**	**0.011**	−0.117	0.624	−0.270	0.249	−0.155	0.527	0.252	0.284	0.396	0.084	0.149	0.529
Concentration of hyaluronic acid (ng/mL)	−0.213	0.368	−0.023	0.924	**−0.606**	**0.005**	**0.605**	**0.005**	**0.602**	**0.005**	**0.742**	**0.000**	0.017	0.942	0.177	0.455	**0.451**	**0.046**

Group 1—patients treated with intravenous thrombolysis; Group 2—patients treated with intravenous thrombolysis and mechanical thrombectomy; Group 3—patients treated with mechanical thrombectomy; T1—before recanalization therapy; T2 and T3—24 h and 48 h after recanalization therapy, respectively. Statistically significant correlations are emphasized with bold formatting.

**Table 3:** Correlations between the concentration of IL-18, NIHSS score, and mRS.

Correlation	Concentration of IL-18 (pg/mL)																	
	Group 1						Group 2						Group 3					
	T1		T2		T3		T1		T2		T3		T1		T2		T3	
	r	*p*	r	*p*	r	*p*	r	*p*	r	*p*	r	*p*	r	*p*	r	*p*	r	*p*
**NIHSS**																		
At admission	−0.241	0.306	−0.140	0.557	−0.211	0.371	**0.520**	**0.025**	**0.544**	**0.020**	**0.565**	**0.018**	−0.073	0.775	−0.075	0.769	0.375	0.125
At discharge	−0.250	0.290	−0.198	0.402	−0.257	0.273	0.117	0.644	0.117	0.645	0.127	0.627	−0.087	0.732	−0.241	0.336	−0.007	0.978
90 days	−0.338	0.145	−0.280	0.230	−0.348	0.133	0.114	0.654	0.156	0.536	0.195	0.453	−0.035	0.891	−0.264	0.291	0.079	0.756
**mRS**																		
At discharge	−0.233	0.324	−0.258	0.273	−0.226	0.337	0.205	0.414	0.198	0.431	0.227	0.308	0.115	0.649	−0.193	0.443	−0.102	0.686
90 days	−0.249	0.290	−0.201	0.395	−0.205	0.385	0.220	0.381	0.250	0.317	0.297	0.247	0.056	0.825	−0.311	0.209	0.004	0.987

Group 1—patients treated with intravenous thrombolysis; Group 2—patients treated with intravenous thrombolysis and mechanical thrombectomy; Group 3—patients treated with mechanical thrombectomy; T1—before recanalization therapy; and T2 and T3—24 h and 48 h after recanalization therapy, respectively. Statistically significant correlations are emphasized with bold formatting.

**Table 4:** Correlations between the concentration of syndecan-1, NIHSS score, and mRS.

Correlation	Concentration of Syndecan-1 (ng/mL)																	
	Group 1						Group 2						Group 3					
	T1		T2		T3		T1		T2		T3		T1		T2		T3	
	r	*p*	r	*p*	r	*p*	r	*p*	r	*p*	r	*p*	r	*p*	r	*p*	r	*p*
**NIHSS**																		
At admission	−0.135	0.570	−0.058	0.808	0.028	0.905	−0.201	0.424	−0.212	0.398	−0.164	0.515	−0.131	0.605	−0.008	0.974	0.290	0.243
At discharge	0.057	0.810	0.301	0.198	0.201	0.395	−0.042	0.868	0.213	0.396	0.098	0.699	−0.170	0.501	−0.273	0.273	0.346	0.160
90 days	−0.038	0.873	0.299	0.209	0.226	0.338	0.084	0.741	0.151	0.551	−0.084	0.740	−0.154	0.542	−0.336	0.173	0.328	0.184
**mRS**																		
At discharge	−0.135	0.571	0.141	0.553	0.200	0.398	−0.018	0.945	0.024	0.924	−0.231	0.356	−0.025	0.923	−0.022	0.932	0.214	0.395
90 days	−0.038	0.872	0.270	0.255	0.267	0.256	0.080	0.753	0.024	0.925	−0.304	0.219	0.031	0.902	−0.145	0.566	0.314	0.204

Group 1—patients treated with intravenous thrombolysis; Group 2—patients treated with intravenous thrombolysis and mechanical thrombectomy; Group 3—patients treated with mechanical thrombectomy; T1—before recanalization therapy; and T2 and T3—24 h and 48 h after recanalization therapy, respectively.

**Table 5:** Correlations between the concentration of heparan sulphate, NIHSS score, and mRS.

Correlation	Concentration of Heparan Sulphate (pg/mL)																	
	Group 1						Group 2						Group 3					
	T1		T2		T3		T1		T2		T3		T1		T2		T3	
	r	*p*	r	*p*	r	*p*	r	*p*	r	*p*	r	*p*	r	*p*	r	*p*	r	*p*
**NIHSS**																		
At admission	**−0.599**	**0.005**	**−0.455**	**0.044**	−0.406	0.076	−0.144	0.569	−0.012	0.963	0.106	0.676	0.415	0.087	0.454	0.059	0.426	0.078
At discharge	**−0.564**	**0.01**	**−0.598**	**0.005**	**−0.629**	**0.003**	0.063	0.804	0.141	0.578	0.193	0.443	0.336	0.172	0.450	0.061	0.357	0.146
90 days	**−0.600**	**0.005**	**−0.635**	**0.003**	**−0.655**	**0.002**	0.066	0.794	0.180	0.475	0.284	0.253	0.191	0.447	0.320	0.196	0.250	0.318
**mRS**																		
At discharge	−0.441	0.051	**−0.450**	**0.047**	**−0.456**	**0.043**	−0.020	0.939	0.383	0.117	0.463	0.053	0.139	0.584	0.319	0.197	0.320	0.195
90 days	**−0.535**	**0.015**	**−0.532**	**0.016**	**−0.514**	**0.020**	−0.147	0.562	0.148	0.558	0.276	0.267	−0.055	0.828	0.147	0.560	0.190	0.451

Group 1—patients treated with intravenous thrombolysis; Group 2—patients treated with intravenous thrombolysis and mechanical thrombectomy; Group 3—patients treated with mechanical thrombectomy; T1—before recanalization therapy; T2 and T3—24 h and 48 h after recanalization therapy, respectively. Statistically significant correlations are emphasized with bold formatting.

**Table 6:** Correlations between the concentration of hyaluronic acid, NIHSS score, and mRS.

Correlation	Concentration of Hyaluronic Acid (ng/mL)																	
	Group 1						Group 2						Group 3					
	T1		T2		T3		T1		T2		T3		T1		T2		T3	
	r	*p*	r	*p*	r	*p*	r	*p*	r	*p*	r	*p*	r	*p*	r	*p*	r	*p*
**NIHSS**																		
At admission	0.054	0.819	−0.122	0.608	**0.600**	**0.005**	0.220	0.381	0.316	0.201	0.249	0.335	−0.197	0.434	0.250	0.317	0.249	0.320
At discharge	0.068	0.774	−0.177	0.455	0.438	0.053	−0.004	0.987	0.025	0.921	0.116	0.659	0.030	0.905	0.146	0.565	−0.020	0.939
90 days	0.098	0.682	−0.260	0.268	**0.493**	**0.027**	−0.022	0.93	−0.023	0.928	0.133	0.610	0.056	0.825	0.172	0.496	0.159	0.528
**mRS**																		
At discharge	0.178	0.453	−0.209	0.377	0.193	0.415	0.018	0.942	0.075	0.768	0.154	0.556	0.153	0.545	0.081	0.749	0.055	0.827
90 days	0.081	0.733	−0.222	0.348	0.210	0.374	0.039	0.880	0.074	0.770	0.185	0.477	0.074	0.770	0.032	0.901	0.134	0.597

Group 1—patients treated with intravenous thrombolysis; Group 2—patients treated with intravenous thrombolysis and mechanical thrombectomy; Group 3—patients treated with mechanical thrombectomy; T1—before recanalization therapy; and T2 and T3—24 h and 48 h after recanalization therapy, respectively. Statistically significant correlations are emphasized with bold formatting.

## Data Availability

The original contributions presented in this study are included in the article. Further inquiries can be directed to the corresponding author.

## Abbreviations

The following abbreviations are used in this manuscript:

BBB	Blood–brain barrier
EG	Endothelial glycocalyx
ELISA	Enzyme-linked immunosorbent assay
eNOS	Endothelial nitric oxide synthase
GAG	Glycosaminoglycan
IL	Interleukin
mRS	Modified Rankin Scale
NIHSS	National Institutes of Health Stroke Scale
NO	Nitric oxide
TICI	Thrombolysis in cerebral infarction
